# Comparing Natural Language Processing and Structured Medical Data to Develop a Computable Phenotype for Patients Hospitalized Due to COVID-19: Retrospective Analysis

**DOI:** 10.2196/46267

**Published:** 2023-08-22

**Authors:** Feier Chang, Jay Krishnan, Jillian H Hurst, Michael E Yarrington, Deverick J Anderson, Emily C O'Brien, Benjamin A Goldstein

**Affiliations:** 1 Department of Biostatistics and Bioinformatics, Duke University, Durham, NC, United States; 2 Department of Medicine, Duke University, Durham, NC, United States; 3 Department of Pediatrics, Duke University, Durham, NC, United States; 4 Department of Population Health Sciences, Duke University, Durham, NC, United States; 5 Duke Clinical Research Institute, Duke University, Durham, NC, United States

**Keywords:** natural language processing, NLP, computable phenotype, machine learning, COVID, coronavirus, hospitalize, hospitalization, electronic health record, EHR, health record, structured data, data element, free text, unstructured data, provider note, classify, classification, algorithm, COVID-19

## Abstract

**Background:**

Throughout the COVID-19 pandemic, many hospitals conducted routine testing of hospitalized patients for SARS-CoV-2 infection upon admission. Some of these patients are admitted for reasons unrelated to COVID-19 and incidentally test positive for the virus. Because COVID-19–related hospitalizations have become a critical public health indicator, it is important to identify patients who are hospitalized because of COVID-19 as opposed to those who are admitted for other indications.

**Objective:**

We compared the performance of different computable phenotype definitions for COVID-19 hospitalizations that use different types of data from electronic health records (EHRs), including structured EHR data elements, clinical notes, or a combination of both data types.

**Methods:**

We conducted a retrospective data analysis, using clinician chart review–based validation at a large academic medical center. We reviewed and analyzed the charts of 586 hospitalized individuals who tested positive for SARS-CoV-2 in January 2022. We used LASSO (least absolute shrinkage and selection operator) regression and random forests to fit classification algorithms that incorporated structured EHR data elements, clinical notes, or a combination of structured data and clinical notes. We used natural language processing to incorporate data from clinical notes. The performance of each model was evaluated based on the area under the receiver operator characteristic curve (AUROC) and an associated decision rule based on sensitivity and positive predictive value. We also identified top words and clinical indicators of COVID-19–specific hospitalization and assessed the impact of different phenotyping strategies on estimated hospital outcome metrics.

**Results:**

Based on a chart review, 38.2% (224/586) of patients were determined to have been hospitalized for reasons other than COVID-19, despite having tested positive for SARS-CoV-2. A computable phenotype that used clinical notes had significantly better discrimination than one that used structured EHR data elements (AUROC: 0.894 vs 0.841; *P*<.001) and performed similarly to a model that combined clinical notes with structured data elements (AUROC: 0.894 vs 0.893; *P*=.91). Assessments of hospital outcome metrics significantly differed based on whether the population included all hospitalized patients who tested positive for SARS-CoV-2 or those who were determined to have been hospitalized due to COVID-19.

**Conclusions:**

These findings highlight the importance of cause-specific phenotyping for COVID-19 hospitalizations. More generally, this work demonstrates the utility of natural language processing approaches for deriving information related to patient hospitalizations in cases where there may be multiple conditions that could serve as the primary indication for hospitalization.

## Introduction

Hospitalization due to COVID-19 has become a key public health indicator. One of the primary goals of vaccination against SARS-CoV-2, the etiological agent of COVID-19, is to reduce the incidence of severe disease and death, with hospitalization serving as a primary end point in vaccine efficacy trials [1]. Further, hospitalization has become a primary indicator of community transmission levels of SARS-CoV-2 infection [2], including disease severity and health system capacity [3-6]. Similarly, hospitalization due to COVID-19 is a typical outcome of interest in public health studies of COVID-19 using real-world data sources, such as electronic health record (EHR) data [7-10]. Finally, because of the rise of rapid, at-home testing for SARS-CoV-2 infection, COVID-19 cases that do not rise to the level of requiring medical attention are likely to be missed or underreported, affecting assessments of COVID-19 prevalence [11]. Thus, there is a critical need to rapidly and accurately identify hospitalizations due to COVID-19.

Due to concerns related to the hospital-based spread of SARS-CoV-2, many institutions routinely perform SARS-CoV-2 testing in patients who are admitted to the hospital, regardless of the primary reason for admission [12,13]. Although SARS-CoV-2 testing is important for guiding care and ensuring that health care professionals take precautions to prevent infection, such routine testing potentially complicates retrospective studies using real-world data sources. Specifically, it becomes challenging to distinguish a patient who was admitted because of COVID-19 from a patient who incidentally tested positive for SARS-CoV-2 infection. In both cases, patients would have a positive laboratory test result and would (presumably) have an *International Classification of Diseases, 10th Revision* (*ICD-10*) code for COVID-19. Previous reports have noted that incidental positives may account for around 26% of all COVID-19–positive patients [14].

Given the public health importance of identifying hospitalizations due to COVID-19 rather than hospitalizations in which SARS-CoV-2 infection was identified incidentally, methods (ie, computable phenotypes) are needed to distinguish the two conditions in retrospective data sources. Such phenotypes would be instrumental in retrospective studies of patients with COVID-19 and in public health surveillance. In this study, we seek to (1) motivate the need to identify patients who were admitted because of COVID-19 versus patients who incidentally tested positive for SARS-CoV-2 during admission, (2) explore the potential of using both structured data (ie, diagnosis codes, medications, and procedure codes) and unstructured data (ie, clinical notes) to construct computable phenotypes, and (3) illustrate the inferential biases that may arise if phenotyping methods cannot distinguish the reason for hospitalization.

## Methods

### Study Setting

We performed a retrospective study of patients aged >18 years who were hospitalized with a documented positive SARS-CoV-2 test result during January 2022. We conducted our study at Duke University Health System (DUHS), which consists of 1 quaternary academic medical center and 2 associated community-based hospitals.

### Ethical Considerations

This study was designated as exempt human subjects research by the DUHS Institutional Review Board (IRB number: Pro00109397).

### Study Data

#### Source Data

Using DUHS EHR data, we identified all patients who were admitted during the week of January 16 to 22, 2022, with documentation of a positive SARS-CoV-2 test result in the prior 20 days. Charts from this week were specifically reviewed in part due to a data request from the North Carolina Division of Public Health to understand the epidemiology of COVID-19–related hospitalizations. We excluded individuals with a resolved COVID-19 isolation status, as well as those who were admitted prior to January 1, 2022, to create a cohort of patients who were likely infected with the Omicron variant of SARS-CoV-2. During this period, the Omicron variant was the predominant SARS-CoV-2 variant in circulation within the United States and was associated with the largest wave [8] of SARS-CoV-2 infections to date. For each patient, we extracted the following data: medical record number, date of admission, hospital unit, and level of care.

To generate a criterion standard for classification, 6 trained health care professionals manually reviewed patient records for the index admission to adjudicate whether SARS-CoV-2 infection was the primary reason for admission or an incidental finding. Health care professionals attributed hospitalizations as those due to COVID-19 if admissions were due to primary manifestations of SARS-CoV-2 infection, such as hypoxia or the need for supplemental oxygen, or due to COVID-19–associated complications, such as dehydration or weakness.

#### Analytic Data

For each admission reviewed, we extracted structured EHR data elements recorded during hospitalization and captured within the Duke Clinical Research Datamart—an EHR database that is based on an extension of the PCORnet Common Data Model (National Patient-Centered Clinical Research Network) [15]. Clinical notes were extracted from the Duke University Electronic Data Warehouse. We extracted admission data, daily progress data, and discharge summary notes. Extracted structured data elements included demographics, service encounter characteristics, diagnoses, laboratory tests, COVID-19 vaccination status, and medications (Table S1 in Multimedia Appendix 1). Clinical notes included emergency department admission notes, progress notes, operative notes, history and physical examination notes, and discharge summaries.

#### Clinical Note Analysis

To analyze the clinical notes, we used the term frequency–inverse document frequency (TF-IDF) approach. The TF-IDF approach [16] generates, across the set of notes for each patient, a numeric value for each word. The word value is based on how common the word is in a patient’s set of notes (term frequency), divided by how common the word is across all of the patient’s notes (inverse document frequency), resulting in a numeric representation for each word on a per-patient basis. Although this is a simple word-based representation, this approach has the following two advantages over deep learning embedding–based approaches: (1) it is possible to directly assess the importance of individual words, and (2) the TF-IDF tends to be more robust with small data sets. Notes were extracted as CSV files and concatenated for the entire encounter. We used the *nltk* package in Python (Python Software Foundation) [17] to tokenize words into a dictionary. For each document, we calculated word counts and removed any words that appeared fewer than 50 times. We then generated the corresponding weight matrix, which served as a numeric input for downstream analyses.

### Analytic Approach

We first described the clinical characteristics of patients hospitalized due to COVID-19 versus those with incidental COVID-19 by using standardized mean differences (SMDs), with an SMD of 0.10 indicating a clinically meaningful difference. Next, we developed 3 classification models for COVID-19–specific hospitalization; one was based entirely on structured EHR data elements, a second was based on clinical notes alone, and a third used both structured data elements and clinical notes. We used LASSO (least absolute shrinkage and selection operator) [18] logistic regression and random forests [19] to estimate the models. Due to the relatively small sample size, we presented our results based on 10-fold cross-validation. We performed the TF-IDF approach separately within each cross-validation fold.

We evaluated the six classification models by calculating the area under the receiver operator characteristic curve (AUROC), along with associated 95% CIs. We identified the top clinical features and words that appeared in clinical notes based on the LASSO and random forest models. We plotted the precision-recall curve to better understand the performance of a classification model and assessed the impact of different rule-based phenotypes.

As a way to understand the importance and potential impact of accurate phenotyping, we performed an illustrative association analysis, evaluating the relationship between vaccination status and the following hospital outcome metrics: length of stay, intensive care unit (ICU) utilization, and in-hospital mortality. These were chosen, since they are standard quality metrics for operational purposes. We regressed each outcome onto vaccination status. We used a log-linear model for length of stay and used logistic regression for ICU utilization and in-hospital mortality. Each regression was performed by using the full cohort and compared to a model that only included patients who were determined to have been hospitalized due to COVID-19. We also tested for an interaction between vaccination status and the cause of hospitalization. We emphasize that these were illustrative analyses, and they were not meant to infer any causal effects of vaccination but rather to illustrate the importance of using cause-specific phenotyping for relevant COVID-19 outcomes.

All work was performed in R version 4.1.2 (R Foundation for Statistical Computing) [20] and Python version 3.9.1 (Python Software Foundation) [21]. The processing code is available in our GitLab (GitLab Inc) [22].

## Results

### Patient Characteristics

In total, we reviewed the charts of 630 patients who were admitted and tested positive for SARS-CoV-2. After excluding patients younger than 18 years and patients with privacy restrictions, our data set included 586 unique patients who were hospitalized and had tested positive for SARS-CoV-2. Of these, 224 (38.2%) were determined, through clinician review, to have been hospitalized for reasons other than COVID-19. During their assessments, our chart reviewers noted that it was often readily apparent which hospitalizations were attributable to COVID-19 and which were not.

Characteristics, by admission cause, are shown in Table 1. Compared with patients hospitalized for indications other than COVID-19, patients hospitalized due to COVID-19 were, on average, older (age: mean 62.7 years vs mean 51.9 years; SMD 0.587), and their admissions were more commonly labeled as emergency admissions (346/362, 95.6% vs 165/224, 73.7%; SMD 0.641). Furthermore, patients hospitalized due to COVID-19 were substantially more likely to receive COVID-19 therapies, including steroids (233/362, 64.4% vs 54/224, 24.1%; SMD 0.887) and the antiviral agent remdesivir (247/362, 68.2% vs 55/224, 24.6%; SMD 0.974), during their hospitalization. Patients hospitalized due to COVID-19 had lower lymphocyte counts on average compared with those of patients hospitalized for reasons other than COVID-19. Normal levels of C-reactive protein and the lack of dimerized plasmin fragment D (D-dimer) testing were associated with hospitalizations for reasons other than COVID-19.

**Table 1.** Cohort description.

Characteristics			Hospitalized due to COVID-19			Standardized mean difference
			No (n=224)	Yes (n=362)	Total (N=586)	
Sex (female), n (%)			120 (53.6)	181 (50)	301 (51.4)	0.072
Age (years), mean			51.9	62.7	58.6	0.587
**Patient outcome at discharge, n (%)**						0.169
	Dead		18 (8)	39 (10.8)	57 (9.7)	
	Home		176 (78.6)	258 (71.3)	434 (74.1)	
	Other facility		30 (13.4)	65 (18)	95 (16.2)	
**Admission type, n (%)**						0.641
	Emergency admission		165 (73.7)	346 (95.6)	511 (87.2)	
	Routine elective admission		24 (10.7)	4 (1.1)	28 (4.8)	
	Urgent admission		35 (15.6)	12 (3.3)	47 (8)	
Transfer to intensive care unit, n (%)			45 (20.1)	78 (21.5)	123 (21)	0.036
**Encounter type, n (%)**						0.181
	Emergency		2 (0.9)	1 (0.3)	3 (0.5)	
	Emergency to inpatient		180 (80.4)	314 (86.7)	494 (84.3)	
	Inpatient		31 (13.8)	35 (9.7)	66 (11.3)	
	Observation stay		11 (4.9)	12 (3.3)	23 (3.9)	
**Race and ethnicity, n (%)**						0.168
	Hispanic		21 (9.4)	20 (5.5)	41 (7)	
	Non-Hispanic Black		106 (47.3)	175 (48.3)	281 (48)	
	Non-Hispanic White		90 (40.2)	152 (42)	242 (41.3)	
	Non-Hispanic Asian		7 (3.1)	14 (3.9)	21 (3.6)	
	Other races		0 (0)	1 (0.3)	1 (0.2)	
Length of stay (days), mean			10.2	9.9	10	0.026
**BMI, n (%)**						0.203
	Missing		9 (4)	9 (2.5)	18 (3.1)	
	Normal		65 (29)	89 (24.6)	154 (26.3)	
	Obese		85 (37.9)	147 (40.6)	232 (39.6)	
	Overweight		60 (26.8)	98 (27.1)	158 (27)	
	Underweight		5 (2.2)	19 (5.2)	24 (4.1)	
**Raw payer type value, n (%)**						0.305
	Private		102 (45.5)	180 (49.7)	282 (48.1)	
	Public		88 (39.3)	144 (39.8)	232 (39.6)	
	Self-pay		21 (9.4)	9 (2.5)	30 (5.1)	
	Other		13 (5.8)	29 (8)	42 (7.2)	
Vaccinated against COVID-19, n (%)			113 (50.4)	178 (49.2)	291 (49.7)	0.026
**Comorbidities, n (%)**						
	Surgery		200 (89.3)	302 (83.4)	502 (85.7)	0.171
	Cancer		29 (12.9)	45 (12.4)	74 (12.6)	0.015
	Cardiovascular		75 (33.5)	146 (40.3)	221 (37.7)	0.142
	Hypertension		73 (32.6)	151 (41.7)	224 (38.2)	0.19
	Chronic liver disease		30 (13.4)	46 (12.7)	76 (13)	0.02
	Chronic obstructive pulmonary disease		21 (9.4)	50 (13.8)	71 (12.1)	0.139
	Asthma		18 (8)	39 (10.8)	57 (9.7)	0.094
	Chronic renal disease		44 (19.6)	111 (30.7)	155 (26.5)	0.256
	Diabetes		45 (20.1)	103 (28.5)	148 (25.3)	0.196
**Medications, n (%)**						
	Bronchodilator		44 (19.6)	159 (41.2)	193 (32.9)	0.481
	Steroid		54 (24.1)	233 (64.4)	287 (49)	0.887
	Anticoagulant antiplatelet		121 (54)	284 (78.5)	405 (69.1)	0.535
	Diuretic		60 (26.8)	131 (36.2)	191 (32.6)	0.203
	Cough suppressant		44 (19.6)	162 (44.8)	206 (35.2)	0.558
	Paralytic		10 (4.5)	30 (8.3)	40 (6.8)	0.157
	Expectorant		14 (6.3)	56 (15.5)	70 (11.9)	0.3
	Remdesivir		55 (24.6)	247 (68.2)	302 (51.5)	0.974
	Inhaled steroid		24 (10.7)	42 (11.6)	66 (11.3)	0.028
**Laboratory tests, n (%)**						
	**Absolute lymphocyte count**					0.345
		High	1 (0.4)	2 (0.6)	3 (0.5)	
		Low	12 (5.4)	47 (13)	59 (10.1)	
		Normal	23 (10.3)	59 (16.3)	82 (14)	
		Not taken	188 (83.9)	254 (70.2)	442 (75.4)	
	**Lymphocyte count**					0.528
		Low	17 (7.6)	71 (19.6)	88 (15)	
		Normal	131 (58.5)	233 (64.4)	364 (62.1)	
		Not taken	76 (33.9)	56 (15.5)	132 (22.5)	
		High	0 (0)	2 (0.6)	2 (0.3)	
	**C-reactive protein**					0.602
		High	62 (27.7)	203 (56.1)	265 (45.2)	
		Normal	11 (4.9)	9 (2.5)	20 (3.4)	
		Not taken	151 (67.4)	150 (41.4)	301 (51.4)	
	**Ferritin**					0.361
		High	39 (17.4)	107 (29.6)	146 (24.9)	
		Low	2 (0.9)	3 (0.8)	5 (0.9)	
		Normal	17 (7.6)	44 (12.2)	61 (10.4)	
		Not taken	166 (74.1)	208 (57.5)	374 (63.8)	
	**D-dimer^a^**					1.187
		High	19 (8.5)	117 (32.3)	136 (23.2)	
		Normal	36 (16.1)	156 (43.1)	192 (32.8)	
		Not taken	169 (75.4)	89 (24.6)	258 (44)	
	**Procalcitonin**					0.524
		High	4 (1.8)	22 (6.1)	26 (4.4)	
		Missing	208 (92.9)	268 (74)	476 (81.2)	
		Normal	12 (5.4)	72 (19.9)	84 (14.3)	

a D-dimer: dimerized plasmin fragment D.

### Performance of Classification Models

After tokenizing words and removing terms with fewer than 50 occurrences, our models included 7953 unique terms. There was minimal difference between the LASSO and random forest models. The random forest model based solely on clinical notes, the one based solely on structured data elements, and the one that used both clinical notes and structured data elements had AUROCs of 0.882 (95% CI 0.85-0.909), 0.829 (95% CI 0.794-0.864), and 0.890 (95% CI 0.864-0.916), respectively. The LASSO model based solely on clinical notes (AUROC=0.894, 95% CI 0.868-0.920) had better discrimination than the LASSO model based solely on structured data elements (AUROC=0.841, 95% CI 0.809-0.874; *P*<.001). The LASSO model using both clinical notes and structured data elements (AUROC=0.893, 95% CI 0.868-0.919) had similar discrimination to that of the LASSO model based solely on clinical notes (*P*=.91).

**Figure 1.**
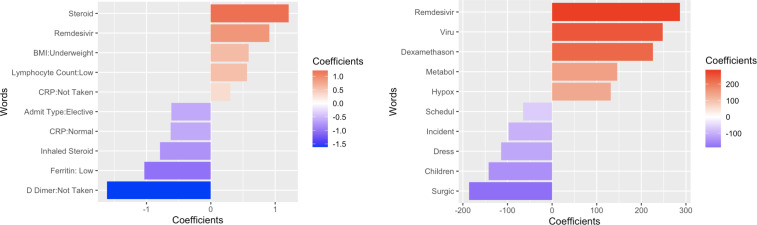
The top regression coefficients from the LASSO models, as reflective of variable importance for (A) the model using just structured data elements and (B) the model using just clinical notes. Values greater than 0 indicate that the feature has a positive association with hospitalization due to COVID-19, while values less than 0 indicate that a feature has a negative association.

Next, we examined the top structured data elements and terms in each model (Figure 1). Highly predictive data elements and words corresponded to patient characteristics with large SMDs (Table 1). Words that are reflective of hospitalization due to COVID-19 have positive coefficients, while words reflective of hospitalization for other reasons have negative coefficients. Terms reflective of COVID-19–specific hospitalization were related to the care of patients with COVID-19, such as “remdesivir” and “dexamethason.” Other structured elements related to the likelihood of being hospitalized for COVID-19 included receipt of steroids, low lymphocyte counts, and underweight BMIs. Terms reflective of hospitalizations due to indications other than COVID-19 included strings that may be related to surgical procedures (eg, “surgic” for “surgical” or “dress” for “dressing”). For structured data elements, a lack of D-dimer collection and low ferritin levels were most commonly associated with admissions for reasons other than COVID-19. Similar features were identified from the random forest model (Figure S1 in Multimedia Appendix 1).

### Impact of Correct Classification

**Figure 2.**
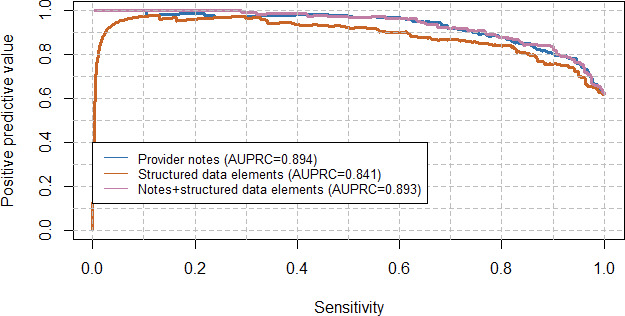
Precision-recall (positive predictive value and sensitivity) curve for the different classification algorithms. This illustrates the trade-off between the identification of patients hospitalized due to COVID-19 (x-axis: sensitivity) and the accuracy of that capture (y-axis: positive predictive value). There is minimal difference between using just notes or notes with structured data elements. The model with only structured data elements performs notably worse in terms of positive predictive value at the same sensitivity thresholds. AUPRC: area under the precision-recall curve.

In order to assess the performance of a computable phenotype–based decision rule, we examined the precision-recall curve of the different models (Figure 2). For example, a rule that maintains a sensitivity of 90% (ie, one that would capture 90% of all patients hospitalized due to COVID-19) resulted in positive predictive values of 76%, 82%, and 84% and corresponding *F*_1_-scores of 0.824, 0.858, and 0.869 based on structured data elements, clinical notes, and their combination, respectively. To illustrate the impact of these differences, we considered the impact of implementing each of these phenotypes at a 90% sensitivity to classify patients during the January Omicron wave. Within our health system, 1378 people were hospitalized and tested positive for SARS-CoV-2. Based on our analyses, using the LASSO-based phenotype that incorporates structured data, clinical notes, or their combination would result in approximately 244, 165, and 142 false positives, respectively.

We next sought to evaluate the potential impact of different phenotyping methods on hospital outcome metrics, comparing a method that incorporates the reason for hospitalization versus one that does not. We used a regression analysis to assess the marginal relationship. As a use case, we evaluated associations between vaccine status and the following three hospital outcome metrics: length of stay, risk of ICU utilization, and in-hospital mortality. These evaluations were performed with the following three cohorts: all hospitalized patients, those who were determined to have been hospitalized due to COVID-19, and those who tested positive for SARS-CoV-2 but were hospitalized for unrelated reasons (Table 2). For length of stay, the magnitude of the effect of vaccine status changed based on the cohort used. In the cohort of all hospitalized patients, vaccinated patients had a shorter length of stay (relative rate 0.81, 95% CI 0.71-0.93). However, when limiting the analytic cohort to patients hospitalized due to COVID-19, there was no significant difference in length of stay for vaccinated patients versus unvaccinated patients (relative rate 0.98, 95% CI 0.83-1.16; *P* value for interaction<.001). We found similar patterns in analyses of other in-hospital outcomes; vaccination was associated with reduced risks of ICU utilization and in-hospital mortality among patients hospitalized for reasons other than COVID-19 when compared to those among patients hospitalized due to COVID-19. Effects were robust to adjustment for age (Table S2 in Multimedia Appendix 1). These results illustrate the impact of selecting the correct cohort for analysis and the potential ramifications of using a cohort in which the reason for hospitalization has not been determined.

**Table 2.** Marginal association between vaccine status^a^ and outcome metrics, unadjusted for age.

Outcome	Full cohort	Hospitalized due to COVID-19	Hospitalization unrelated to COVID-19	*P* value^b^
Length of stay, relative rate (95% CI)	0.81 (0.71-0.93)	0.98 (0.83-1.16)	0.59 (0.47-0.74)	<.001
ICU^c^ utilization, odds ratio (95% CI)	1.04 (0.70-1.56)	1.25 (0.75-2.07)	0.77 (0.40-1.49)	.26
Mortality, odds ratio (95% CI)	1.02 (0.59-1.78)	1.45 (0.74-2.88)	0.48 (0.16-1.29)	.08

a Unvaccinated patients are the reference group.

b *P* value is for hospitalization due to COVID-19 versus hospitalization unrelated to COVID-19.

c ICU: intensive care unit.

## Discussion

### Principal Findings

Due to the public health importance of the accurate identification of COVID-19–related hospitalizations, there is a need for methods and computable phenotypes to identify hospital admissions in which the primary cause is COVID-19 [23]. We used machine learning methods and a physician chart review to develop a classification algorithm for hospitalization due to COVID-19. We found that 38.2% (224/586) of patients who were hospitalized at our institution during the Omicron wave and tested positive for SARS-CoV-2 infection were hospitalized for reasons other than COVID-19. These findings are in line with other recent studies, which found that an average of 26% of hospitalized patients with a positive SARS-CoV-2 test result had a primary indication for hospitalization that was unrelated to COVID-19 [14]. We found that a model based on clinical notes performed better than one based solely on structured EHR data elements. This work has important implications for retrospective analyses using EHR data to assess outcomes related to COVID-19, including vaccine effectiveness and health system capacity [24].

Prior work by Lynch and colleagues [25] evaluated the utility of *ICD-10* codes for COVID-19 diagnosis in inpatient, outpatient, emergency care, and urgent care settings during time periods across the pandemic; using a weighted, random sample of 1500 records from the Department of Veterans Affairs, they found that the COVID-19 *ICD-10* code (U07.1) had a relatively low positive predictive value across settings and time periods. These findings highlight the need for additional contextual data to identify acute cases of COVID-19. The Consortium for Clinical Characterization of COVID-19 by EHR (4CE) conducted a similar study of EHR data from 12 clinical sites to identify combinations of structured data elements to generate a reliable computable phenotype for hospitalization due to COVID-19, with a reported AUROC of 0.903 [26]. Similarly, we derived an AUROC of 0.841 based solely on structured data elements; however, we also found that that inclusion of clinical notes significantly improved the performance of the classification model (AUROC=0.893; *P*<.001). This result is not surprising, as the clinical narrative often includes important nuance, and as our chart reviewers noted, it was often readily apparent which hospitalizations were attributable to COVID-19 and which were not. Of note, chart reviewers in our study classified hospitalizations that were indirectly due to SARS-CoV-2 infection, such as those due to COVID-19–related weakness or delirium, as hospitalizations due to COVID-19, which could partly explain the observed difference in discriminatory ability between our study and the study conducted by the 4CE.

By using the TF-IDF approach in conjunction with LASSO regression, we identified both individual terms and the direction of the association between each term and the hospitalization indication. Although the TF-IDF approach is a simple natural language processing (NLP) approach, it is also very scalable, interpretable, and implementable. Our results highlight the power of even simple natural language models. The terms that best predicted hospitalizations due to COVID-19 included common descriptors that were used in the clinical care of patients with COVID-19, such as “hypox” (likely shortened from “hypoxia” or “hypoxic”), or COVID-19 therapies like remdesivir. Conversely, the terms that were not associated with hospitalizations due to COVID-19 included words related to surgery—a common indication for hospital admission that is generally unrelated to SARS-CoV-2 infection.

To help contextualize our results, we also assessed the real-world impact of using an accurate phenotype for COVID-19–specific hospitalization. In studying hospitalized patients with COVID-19, the simplest analysis would be to include all patients with a COVID-19–positive test result. As our illustrative analysis showed, when using this full but heterogeneous cohort, the results suggested that vaccination status is associated with a shorter length of stay. However, when we limited the analysis to only include patients who were identified as having been hospitalized due to COVID-19 (ie, people with symptoms of COVID-19), the analysis indicated that vaccines are not associated with a shorter length of stay. We interpreted these data as indicating that, conditional on someone being sick enough to be hospitalized due to COVID-19, vaccines provide no additional benefit in terms of the length of hospitalization. Similar patterns were found for other hospital outcome metrics. Although this analysis was not intended to be a causal analysis, it did illustrate how the use of accurately classified cohorts is important for the calculation of standard outcome metrics and likely impacts other related association analyses.

More broadly, this work highlights the importance and challenge of phenotyping cause-specific events. Although there is rich literature on computable phenotypes, most of this literature is geared toward the identification of chronic diseases (eg, presence of asthma). However, few computable phenotypes have focused on cause-specific events (eg, asthma exacerbation). Such cause-specific phenotypes often exhibit poor specificity and can require algorithms that are more complex than those required for chronic conditions. As this work shows, and as suggested by others, NLP-based phenotyping approaches are becoming more common, and further comparisons between NLP approaches and other methods will be needed to determine whether using text data can improve cause-specific phenotypes.

Although our study used rigorous methods, there are some key limitations. First and most notably, our findings are primarily illustrative and may not represent a generalizable algorithm for phenotyping COVID-19–specific hospitalizations. This study was conducted across a single hospital system, and it may not be reflective of practices at other institutions. Importantly, we would not expect our specific phenotype algorithm to be generalizable to other institutions. Second, we only looked at 1 period of time, namely the January 2022 Omicron wave; however, there are documented differences in the rate of hospitalization and positive test results over the course of the pandemic, and our models may not accurately reflect distinguishing factors in other waves. Third, another limitation is that, given the time constraints of chart reviews, we were only able to analyze a relatively small sample. In particular, the small sample size limited our ability to apply more sophisticated NLP-based approaches, such as the use of n-grams.

### Conclusions

Overall, our results show that a sizable number of people who were hospitalized and tested positive for SARS-CoV-2 were hospitalized for reasons other than COVID-19. The conflation of these individuals can impact our understanding of hospital outcome metrics. We constructed a strong classification model that can be used as a computable phenotype to distinguish patients who were hospitalized due to COVID-19 from those who incidentally tested positive for SARS-CoV-2 but were hospitalized for other reasons. Moreover, we found that while structured data elements are useful in constructing such a phenotype, clinical notes had a higher positive predictive value than that of structured data elements alone. Future work should seek to explore the generalizability of such phenotypes across institutions and different waves of the COVID-19 pandemic.

## Supplementary material

10.2196/46267 Multimedia Appendix 1 Supplemental materials regarding variable descriptions, top data elements, and the association between vaccine status and outcome metrics.

## Abbreviations

4CE: Consortium for Clinical Characterization of COVID-19 by EHR
AUROC: area under the receiver operator characteristic curve
D-dimer: dimerized plasmin fragment D
DUHS: Duke University Health System
EHR: electronic health record
*ICD-10*: *International Classification of Diseases, 10th Revision*
ICU: intensive care unit
LASSO: least absolute shrinkage and selection operator
NLP: natural language processing
SMD: standardized mean difference
TF-IDF: term frequency–inverse document frequency
