# Supplementary material for: Comparing Natural Language Processing and Structured Medical Data to Develop a Computable Phenotype for Patients Hospitalized Due to COVID-19: Retrospective Analysis
Source: JMIR Med Inform. 2023 Aug 22;11:e46267. doi: 10.2196/46267 (PMC10466442; doi:10.2196/46267)
Supplement: Multimedia Appendix 1 [file medinform-v11-e46267-s001.docx]

**Supplemental Material**

**Table S1:** Variable Description

| Grouping | Variable | Definition |
| --- | --- | --- |
| Demographic | Sex |  |
|  | Age |  |
|  | Race |  |
|  | BMI | Category: Underweight, Normal, Obese, Overweight |
| Medical information | Patient outcome at discharge | Describe where the patient was discharged after an inpatient or emergency department encounter. |
|  | Admission Type | The description of the admission type (elective surgery, etc.). |
|  | Transfer to ICU | Indicates whether the patient was ever transferred to the ICU during an inpatient stay. |
|  | Encounter Type | Category: Emergency Department, Emergency Department Admit to Inpatient Stay, Inpatient Hospital Stay, Observation Stay |
|  | Length of Stay | Discharge Date minus Admit Date |
|  | Raw payer type value | Payment Information  Category: Private(commercial, managed care, medicare advantage, nc blue cross, oos blue cross), Public(medicaid pending, medicare, other government, nc medicaid, nc medicaid managed care), Self-pay, Other(special programs) |
|  | Vaccine Status | COVID Vaccine Status |
| Comorbidity | Cancer | ICD-10 code: C* |
|  | Cardiovascular | ICD-10 code: I20-I59 |
|  | Hypertension | ICD-10 code: I10-I11 |
|  | Chronic Liver Disease | ICD-10 code: K70-K77 |
|  | Chronic Obstructive Pulmonary Disease | Chronic Obstructive Pulmonary Disease  ICD-10 code: J44 |
|  | ASTHMA | Asthma  ICD-10 code: J45 |
|  | Chronic Renal Disease | ICD-10 code: N18 |
|  | Diabetes | ICD-10 code: E08-E13 |
| Medicine | Bronchodilator | Bronchodilator |
|  | Steroid | Steroid, Corticosteroid |
|  | Anticoagulant Antiplatelet | Anticoagulant |
|  | Diuretic | Diuretic |
|  | Cough Suppressant | Cough Suppressant,  Expectorant with cough suppressant |
|  | Paralytic | Paralytic,  Used during intubation as a paralytic |
|  | Expectorant | Expectorant |
|  | Remdesivir | Remdesivir |
|  | Inhaled.Steroid | Inhaled Steroid |
| Lab test | Lymphocyte.Count..Absolute | The absolute number of Lymphocytes |
|  | Lymphocyte.Count | The number of Lymphocytes |
|  | CRP..C.reactive.Protein..Inflammatory | C-Reactive Protein |
|  | Ferritin | Ferritin level: High, Low, Normal, Not Taken |
|  | D.Dimer | D-Dimer level: High, Normal, Not Taken |
|  | Procalcitonin | Procalcitonin level: High, Missing, Normal |

**Figure S1**


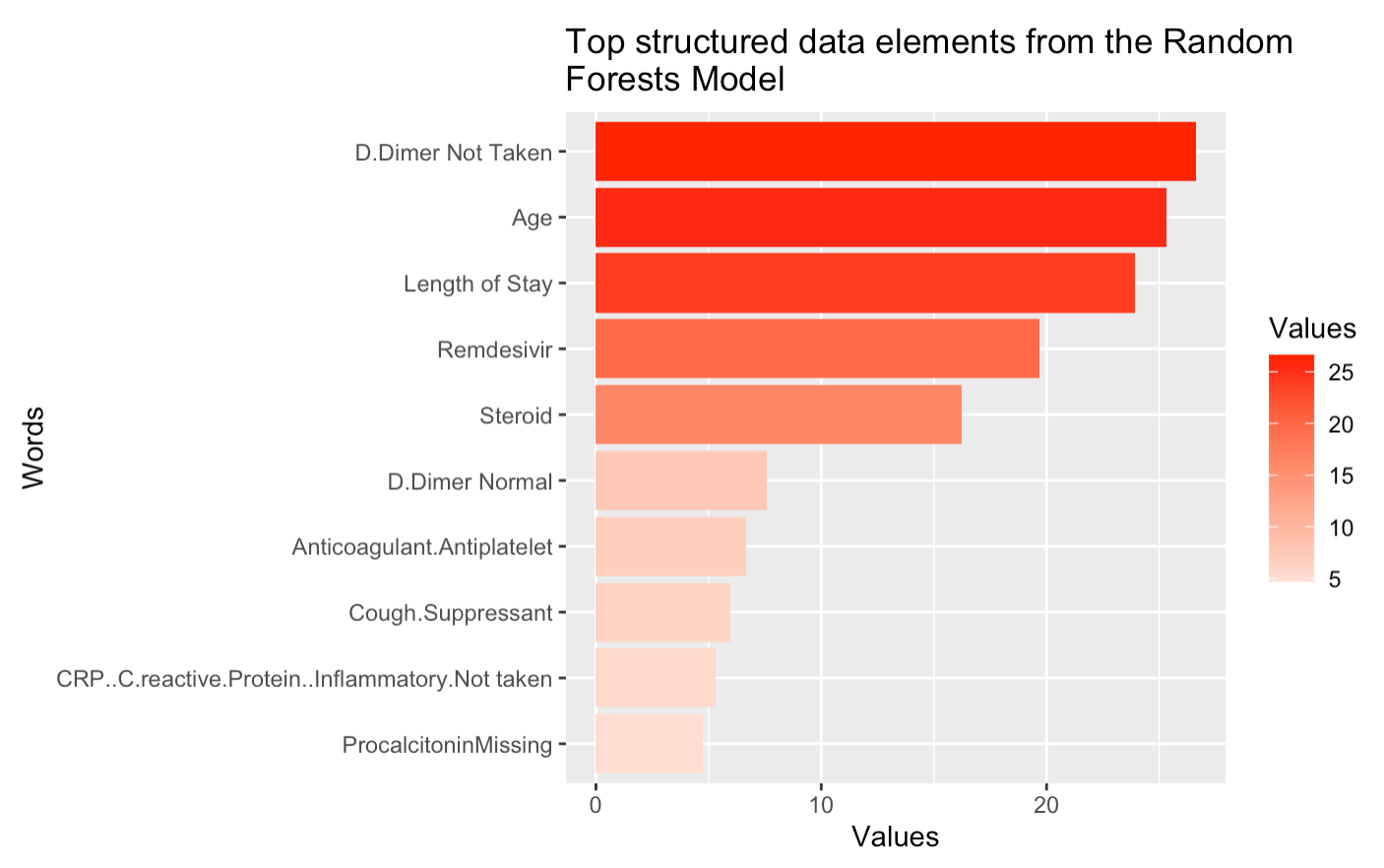


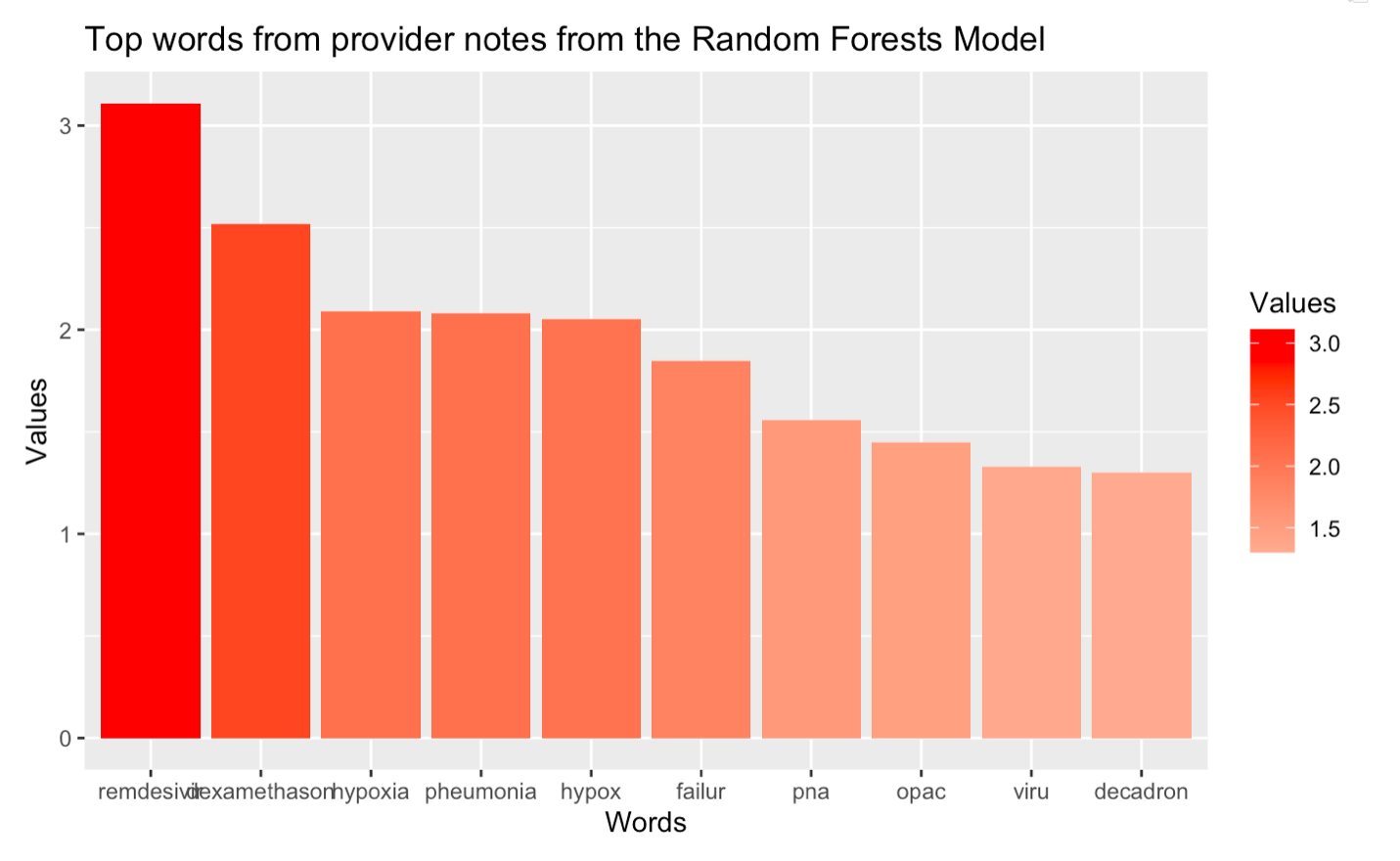


**Legend**: Top structured clinical data elements (a) and words from notes (b) detected by the Random Forests model. The top features do not imply any directionality of effect.

**Table S2:** Association between vaccine status and outcome metrics adjusted for age

| Marginal association between vaccine status* and outcome metrics, unadjusted for age **Outcome** | **Full Cohort (95% CI)** | **Hospitalized Due to COVID-**  **19 (95% CI)** | **Hospitalization unrelated to COVID-19 (95% CI)** | **P-value Hospitalization due to COVID-19 vs. Hospitalization unrelated to COVID-19** |
| --- | --- | --- | --- | --- |
| LOS | 0.87(0.76 0.99) | 1.02(0.86 1.20) | 0.67(0.54 0.84) | 0.001 |
| ICU | 1.12(0.75 1.65) | 1.30(0.78 2.17) | 0.87(0.44 1.71) | 0.328 |
| Mortality | 1.27(0.72 2.25) | 1.69(0.85 3.46) | 0.68(0.22 1.92) | 0.172 |

* Unvaccinated patients are the reference group.
